# Supplementary material for: Multi-omics profiles of chronic low back pain and fibromyalgia—Study protocol
Source: PLoS One. 2025 Apr 16;20(4):e0312061. doi: 10.1371/journal.pone.0312061 (PMC12002477; doi:10.1371/journal.pone.0312061)
Supplement: Attachment — Submitted filename: Reply to reviewers - FIBMET protocol.docx [file pone.0312061.s002.docx]

January 21, 2025

Editorial Board, PLOS ONE

**RE: Ms. No. PONE-D-24-43124**

**Multi-Omics Profiles of Chronic Low Back Pain and Fibromyalgia – Study Protocol**

Dear Dr. Sommer

On behalf of the authors, I would like to thank you and the reviewer for the interest in our protocol and the very constructive comments, which have been very helpful to improve the paper. Please find our responses below. We hope that the revision will address the comments satisfactorily.

Yours sincerely

Michele Curatolo, MD, PhD

Professor of Anesthesiology and Pain Medicine

Endowed Professor in Medical Education and Research

**Reviewer #1:**

The PLOS Data policy requires authors to make all data underlying the findings described in their manuscript fully available without restriction, with rare exception, at the time of publication. The data should be provided as part of the manuscript or its supporting information, or deposited to a public repository. For example, in addition to summary statistics, the data points behind means, medians and variance measures should be available. If there are restrictions on publicly sharing data—e.g. participant privacy or use of data from a third party—those must be specified.

Reply: We now make the following statement:

*No datasets were generated or analyzed during the current study. All relevant data from this study will be made available upon study completion and publications.*

Please provide an appropriate reporting guideline according to which you have compiled your study protocol and also provide the appropriate checklist with the revised manuscript.

Reply: We have not followed a specific guideline to compile the study protocol.

I could not identify as to whether your study protocol was pre-registered in an appropriate registry or as to whether you planned to do so; if not, please consider to do so and provide information in the revised manuscript accordingly.

Reply: Because the study is not a clinical trial, we have not pre-registered it. At this time, we can no longer register the study because the data collection has started, and most journals do not accept registrations that are done during the data collection.

Introduction: Please provide a paragraph detailing potential a-priori hypotheses you might have based on your previous research findings.

Reply: Because of the very limited previous research, we do not have *a priori* hypotheses that involve specific molecular pathways. As explained in the section “Data analysis”, we will adopt a holistic approach to evaluate the association between omics profiles and phenotyping that will consider convergent associations with multiple pain-related phenotyping domains.

Changes in the manuscript: We have added the following text at the end of the introduction:

*Because of the very limited previous research, we do not have an a priori hypothesis involving specific molecular pathways, and will perform unbiased analyses to allow discovery of potentially relevant molecular pathways and candidate biomarkers.*

Table 1: Currently, potential participants are 'only' screened for cancer as a 'red flag' exclusion criterion. However, there may be additional red flags that need to be addressed, e.g. (osteoporotic) fractures, cauda equine syndrome, systemic inflammation etc.. Please provide additional information if or where the protocol intends to screen for additional red flags or what additional measures will ensure that the conditions are CLBP and/or FM in nature.

Reply: Thank you for your comment. We do not exclude patients with other pain syndromes, as their presence would not rule out *per se* CLBP or fibromyalgia, according to the diagnostic criteria of the International Association for the Study of Pain (IASP) and American College of Rheumatology, respectively (see section “Participants and eligibility criteria”, 2^nd^ paragraph). However, we agree that the presence of some conditions could influence the omics profile. As explained in the section “Phenotyping measures”, we will record any pain diagnosis and analyze potential outliers for diagnoses that could influence the omics profiles.

Changes in the manuscript: We have added the following test to the section “Data analysis”, 4^th^ paragraph:

*Sensitivity analyses will be performed to study the potential influence of outliers, such as patients with unusual pain diagnoses.*

We recognize that “unusual diagnoses” may seem vague. However, we believe that this approach may provide the opportunity for potential discoveries in patients with unusual findings. This has proven useful in previous studies of our group, see <https://doi.org/10.1101/2024.11.12.24317004>.

Neuropathic components: People will be screened for potential neuropathic components by various measures; if people are shown to have neuropathic pain components, will they be treated as a subset of CLBP, as the underlying pain mechanisms are different from those currently discussed in the context of CLBP? Therefore, please provide additional information on whether people presenting with neuropathic pain components will be treated as a subgroup and separately from the CLBP group.

Reply: As mentioned in the section “Data analysis”, last paragraph of page 13:

*We will associate multi-omics profiles to pain-related clinical characteristics, physical function, indicators of neuropathic pain, and pain facilitation. We anticipate identifying omics profiles and pathways that are associated with pain phenotypes. This will provide information on mechanisms of specific importance to CLBP and FM that are linked with specific clinical pain phenotypes.*

We hope that this will address your comment. See also reply to your comment below regarding neuropathic components.

Control group neuropathic component testing: Please provide more detailed information on the definition of "sensory abnormalities" based on the QST results of the testing of this group.

Reply: Thanks for your comment.

Changes in the manuscript: We have modified the text as follows (bottom of page 10):

*Testing the control group will allow the determination of sensory abnormalities in patients, specifically reduced sensation with different stimulation modalities and pain to innocuous stimuli (allodynia), as recommended by diagnostic guidelines for neuropathic pain (23, 46, 60, 61).*

Please provide more detailed information on the determination of “pain facilitation” based on the temporal summation results of the testing of this group.

Changes in the manuscript: We have rephrased and added text to made it clearer (section “Pain facilitation” on page 12):

*Temporal summation refers to an increased perception of pain in response to repeated stimuli of equal intensity, indicating facilitation of pain modulation processes (65-67). Temporal summation is enhanced in CLBP and FM (35, 36, 48, 49). We use a pinprick stimulator at 256 mN. Patients first rate the intensity of a single stimulus (0 = no pain, 100 = worst pain imaginable). We then apply a series of 10 identical stimuli with a frequency of 1 Hz using 2 Hz metronome ticks as a guide, with patients rating the pain intensity at the end of the series. This sequence is applied at the same three sites as the QST. Pain facilitation is assessed as the difference between pain rating after 10 stimuli and pain rating after 1 stimulus: the higher the difference, the higher the facilitation of pain modulation processes (25).*

Data analysis: Against the aspects mentioned before, please explain as to whether the sub-group of patients demonstrating neuropathic pain components could also/could not present an additional dependent variable in the multinomial regression analysis.

Reply: We agree. However, we anticipate that neuropathic pain indicators will represent a continuum, rather than determine a subgroup of patients. On page 14, 2^nd^ paragraph, we state: “*In exploratory analyses, we will perform linear, logistic, and multinomial logistic regressions for continuous, binary, and categorical dependent variables, respectively, with the phenotyping measures as dependent variables.*” The phenotyping measures include the indicators of neuropathic pain as outlined on page 10. We hope that this addresses your question.

Discussion: As mentioned by the authors and reflected in the Methods section, this study is exploratory in nature. Therefore, the results will provide associations between multi-omics profiles and pain-related clinical features; however, these associations require further investigation before they can serve as "converging evidence for potential targets for future therapeutic development". Please be more cautious in this conclusion and provide a more detailed description of how the results could inform future research.

Changes in the manuscript: We agree that the study will not deliver targets for immediate pharmacological development. Since the word “potential” may not be cautious enough, and according to your request on how the results can inform future research, we have added the following text (last paragraph of the Discission): *“The results will inform future hypothesis-driven mechanistic studies to identify relevant targets for pharmacological development, as well as biomarker validation studies.”*

Additional not-requested change: We realized we had omitted an author, Savera Kahn, now added.
